# Reconstructing mammalian lifespan evolution reveals strong phylogenetic effects and lifespan-associated genes

**DOI:** 10.1186/s12915-026-02599-3

**Published:** 2026-04-17

**Authors:** Zhen-Na Zhang, Xue Lyu, Shan-Zhuang Niu, Dong-Ming Xu, Cheng-Gang Zou

**Affiliations:** 1 State Key Laboratory for Conservation and Utilization of Bio-Resources in Yunnan, School of Life Sciences, Yunnan University, Kunming, China; 2 State Key Laboratory of Genetic Resources and Evolution, Kunming Institute of Zoology, Chinese Academy of Sciences, Kunming, Yunnan China

**Keywords:** Mammal, Longevity, Bat, *C. elegans*

## Abstract

**Background:**

Despite the extraordinary diversity in mammalian lifespans, the evolutionary trajectories and underlying molecular mechanisms governing this variation remain largely uncharacterized.

**Results:**

By reconstructing maximum lifespan evolution across 968 mammalian species, we found that ~ 23 lineages evolved relatively longer lifespans, while ~ 25 evolved relatively shorter lifespans. To understand the phylogenetic influence on lifespan evolution, we tested 9 evolutionary models and found that Pagel’s lambda was the most suitable, indicating a strong effect of shared evolutionary histories on the evolution of mammalian lifespans. Through comparative genomic analysis of 15,231 one-to-one ortholog genes across 122 mammalian species, we identified 628 genes associated with variation in relative lifespan (i.e., longevity quotient). Genes whose evolutionary rates negatively correlated with relative lifespans were enriched for functions related to cell division and DNA repair, whereas those with positive correlations were primarily involved in ion transport and muscle contraction. In vivo experiments further validated the functional relevance of 11 candidate genes in *C. elegans*. In particular, inhibition of the *phi-53* gene extended lifespan, which was associated with upregulation of genes enriched in immune-related functions and downregulation of genes enriched in reproduction-related functions.

**Conclusions:**

Our findings highlight the crucial role of phylogenetic history in shaping mammalian lifespan evolution and provide important molecular insights into the mechanisms governing lifespan variations in mammals.

**Supplementary Information:**

The online version contains supplementary material available at 10.1186/s12915-026-02599-3.

## Background

Lifespan is among the most variable life-history traits in mammals, ranging more than 100-fold, from as little as two years in the forest shrew (*Myosorex varius*) to over 200 years in the bowhead whale (*Balaena mysticetus*) [1, 2]. Multiple factors have been proposed to explain this extensive variation, including extrinsic mortality, life-history strategy (pace of life), metabolism, and body size [3, 4]. For instance, flight reduces predation risk and is associated with exceptional longevity in bats compared to similarly sized non-flying mammals [5]. Species positioned at the “slow” end of the fast–slow continuum tend to mature later, produce smaller litters, invest more heavily in individual offspring, and have longer lifespans, while “fast” species show the opposite pattern [6]. In general, lifespan in mammals is inversely correlated with mass-specific metabolic rate [7]. Social organization also plays a role: group-living species generally live longer than solitary species, suggesting a link between sociality and longevity [8]. Despite these contributing factors, a general observation is that lifespan is positively correlated with body mass among mammals [9]. Larger species usually have lower mass-specific metabolic rates, reduced predation, slower reproductive rates, and longer maximum lifespans [10, 11]. These factors, which arose during mammalian evolution, are associated with the wide variation in lifespan observed across mammalian species and have complicated our understanding of how phylogenetic relationships among species influence lifespan evolution.

Despite the substantial variation of mammalian lifespans, several previous studies have sought to identify genes closely associated with lifespan differences across species. For example, genes involved in DNA repair, the mTOR pathway, insulin signaling, and the p53 signaling pathway have been found to undergo positive selection or convergent evolution in long-lived mammals, indicating their important roles in lifespan regulation [1, 2, 9, 12–15]. Although these studies have significantly advanced our understanding of the ecological, physiological, and molecular underpinnings of mammalian lifespans, how lifespan changes along different branches of the entire mammalian phylogeny and the selective pressures acting on lifespan-related genes remain unclear. Although previous studies have also investigated lifespan evolution, most focused either on specific mammalian clades, such as primates [13] and bats [5, 16], or on a relatively limited set of species [1]. Elucidating these evolutionary dynamics is crucial for identifying the genetic changes that influence mammalian lifespans by linking patterns of gene evolution to variation in lifespan traits.

To address these questions, we evaluated evolutionary patterns of mammalian lifespans using the largest taxonomic sampling to date. Our analyses revealed that phylogenetic signals have a substantial impact on the evolution of mammalian lifespans, and that Pagel’s λ was the most suitable model for this evolution. We further found that both intensified and relaxed evolutionary pressures have played important roles in shaping lifespan diversity among mammals, indicating that lifespan differences did not arise from a single evolutionary process. In some mammalian lineages, natural selection acted more strongly on lifespan, whereas in others, selection was weaker, allowing lifespan to change more freely, become less constrained, or evolve in different directions. These findings offer new insights into the evolutionary dynamics of mammalian lifespans and improve our understanding of the molecular mechanisms underlying lifespan variations in mammals.

## Results

### Evolutionary patterns of mammalian lifespan

**Fig. 1:**
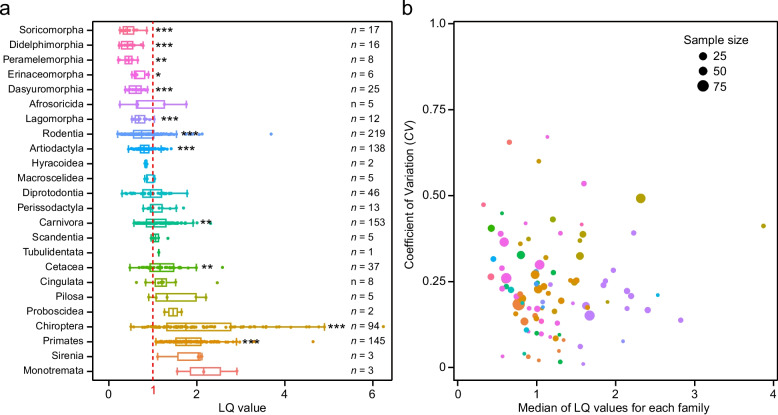
Diversity and variance of relative lifespans across 968 mammalian species. **a** The distribution of relative lifespans (LQ) among various mammalian taxonomic groups is presented at the order level. For each order, the first and third quantiles, along with the median LQ values, are depicted to illustrate the range and central tendency of lifespans. *n* represents the species number within each order. The *P* values are from binomial tests and are adjusted using the false discovery rate (FDR). *P < 0.05, **P < 0.01, *** *P* < 0.001. **b** The relationship between median LQ and its variance across different families in mammals. The sample size indicates the number of species for each mammalian family

To understand how lifespans are distributed across mammals, we collected lifespan data of 968 mammalian species spanning 24 orders and 123 families (Additional file 1: Table S1) from the AnAge database [17]. Since lifespan is highly correlated with body mass, we normalized lifespan values using the longevity quotient (LQ; see the “ Methods” section for details). In our dataset, mammalian lifespans varied by nearly 31-fold, with LQ values ranging from 0.2 to 6.24 (Fig. 1a; Additional file 1: Table S1). Some mammalian orders exhibited distinctly longer or shorter lifespans than expected lifespans, which were estimated using a least-squares regression formula (see the “ Methods” section for details). Orders with the highest median LQ values—such as Primates (1.754 ± 0.501; mean ± SD), Chiroptera (1.748 ± 1.084), and Cetacea (1.166 ± 0.401)—demonstrated significantly higher observed lifespans than predicted (adj-*P* < 0.01; binomial tests; Fig. 1a; Additional file 1: Table S2), marking them as relatively long-lived groups. Conversely, Soricomorpha (0.420 ± 0.160), Didelphimorphia (0.425 ± 0.184), Peramelemorphia (0.452 ± 0.148), Erinaceomorpha (0.615 ± 0.161), and Dasyuromorphia (0.619 ± 0.160) had the lowest median LQs, indicating significantly shorter observed lifespans (adj-*P* < 0.05), and thus represent relatively short-lived groups. At the species level, the Bennett’s chinchilla rat (*Abrocoma bennettii*, order: Rodentia) showed the shortest relative lifespan (LQ = 0.201 vs. median LQ = 0.74 within Rodentia), while the Brandt’s bat (*Myotis brandtii*, Chiroptera) had the longest (LQ = 6.238 vs. median LQ = 1.75 within Chiroptera). We further analyzed variation within families using the coefficient of variance (*CV*; Fig. 1b; Additional file 1: Table S3), indicating that most families (83.9%)—whether generally long-lived or short-lived—exhibited high relative lifespan variation (*CV* > 0.1). This indicates that large lifespan diversity exists within families, independent of their overall longevity tendencies. Notably, because LQ values are limited in some mammalian families, the availability of additional LQ data in the future may affect the precise comparisons of variance in relative lifespans across families.

To investigate whether phylogenetic history shapes mammalian lifespan diversity, we fit several commonly-used models of trait evolution using the “geiger” *R* package [18] based on a 968-species phylogeny from the TimeTree database [19]. The models tested included Brownian motion, Pagel’s λ, trend diffusion, delta, kappa, Ornstein–Uhlenbeck, early-burst, drift, and a white-noise (non-phylogenetic) model. Our analysis showed that all evolutionary models fit the mammalian LQ values better than the white-noise model (*P* < 0.001; *χ*^2^ tests; Table 1), indicating that mammalian relative lifespans are strongly shaped by evolutionary relationships. Of all the models tested, Pagel’s λ provided the best fit for explaining variation in mammalian LQ values, as indicated by the highest likelihood and the lowest Akaike Information Criterion (AIC) values (Table 1). This suggests that shared evolutionary history is the primary driver of LQ distributions at the tips of the mammalian phylogeny. The λ value, which measures the strength of phylogenetic relationships in predicting trait variation at the tips of a phylogeny [20], was estimated to be 0.96, suggesting that the evolution of mammalian LQ values closely follows the expected phylogenetic signal under the Brownian motion model, despite significant differences between the two models (*P* < 0.0001, *χ*^2^ test). Additionally, estimates for kappa (κ = 0.61) and delta (δ = 3.00) parameters, reflecting the mode and tempo of trait evolution [21], suggest that mammalian LQ values have evolved mostly gradually along ancestral branches, with episodes of lineage-specific, accelerated change at the tips. Notably, when we performed parallel evolutionary analyses of absolute maximum lifespan data in mammals, Pagel’s λ also emerged as the best model (Additional file 1: Table S4), reinforcing our conclusions from the LQ analysis.

**Table 1:** Summary of model fitting for the evolution of mammalian lifespans

Model	Log-likelihood^a^	Number of parameters	Parameters	AIC	*P* value^b^
Brownian motion	− 585.44	2	Sigma = 0.015, root state = 1.328	1174.88	< 0.0001
Ornstein–Uhlenbeck	− 542.83	3	Alpha = 0.022, sigma = 0.015, root state = 1.185	1091.65	< 0.0001
Early-burst	− 585.44	3	Alpha = 0.000, sigma = 0.022, root state = 1.328	1176.89	< 0.0001
**Pagel’s model**	**− 497.19**	**3**	**Alpha = 0.964, sigma = 0.007, root state = 1.326**	**1000.38**	**< 0.0001**
Trend diffusion (rate trend)	− 574.22	3	Slope = 99.998, sigma = 0.000, root state = 1.248	1154.45	< 0.0001
Kappa	− 535.16	3	Kappa = 0.601, sigma = 0.027, root state = 1.396	1076.32	< 0.0001
Delta	− 564.98	3	Delta = 3.000, sigma = 0.005, root state = 1.212	1135.97	< 0.0001
Drift	− 584.92	3	Drift = − 0.119, sigma = 0.015, root state = 22.794	1175.84	< 0.0001
White-noise	− 1000.37	2	Sigma = 0.463, root state = 1.161	2004.74	

^a^The logarithm of the likelihood value

^b^The *P* values are from the chi-square tests when compared with the white-noise model

The boldface highlighted the best model

**Fig. 2:**
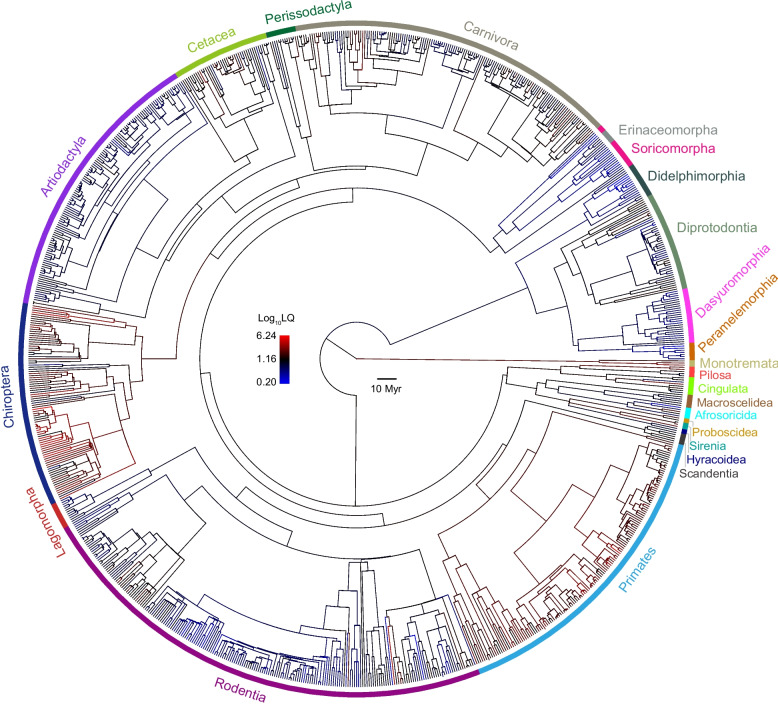
Ancestral state reconstruction of longevity quotient (LQ) for 968 mammalian species. Lineages with LQ values below 0.4 were classified as exceptionally short-lived (shown in blue), while those with LQ values above 2.4 were classified as exceptionally long-lived (shown in red)

To reconstruct the evolutionary trajectories of mammalian relative lifespans, we used a maximum-likelihood method [22] to estimate the ancestral LQ states across the phylogeny of 968 mammalian species [19]. As the distribution of LQ values from these species is bell-shaped, and log-transformed LQ values closely follow a normal distribution (Additional file 2: Fig. S1), we arbitrarily used threshold LQ values of 0.44 and 2.40 to define exceptionally relatively short-lived and long-lived lineages, respectively, corresponding to the lowest and highest 5% of species in our dataset. Based on these thresholds, our ancestral reconstructions identified at least 25 lineages with relatively short lifespans and at least 23 with relatively long lifespans (Fig. 2). To assess the reliability of these ancestral-state reconstructions, we reanalyzed the data using BayesTraits [23]. This Bayesian analysis identified at least 28 lineages with relatively short lifespans and at least 19 lineages with relatively long lifespans (Additional file 2: Fig. S2).

Both highly consistent results strongly support the repeated evolution of short-lifespans and long-lifespans in mammals. Our analyses also showed that the evolution of short lifespans was phylogenetically widespread across mammals, occurring in multiple, distantly related lineages. In contrast, the evolution of long lifespans appears to be more phylogenetically restricted, with most long-lived species clustered within specific groups such as primates and bats. This pattern is consistent with previous studies, showing that longevity has evolved in only a few mammalian clades, notably among bats and primates, while short lifespans are distributed across a broad phylogenetic range [5, 24]. Such clustering of long-lived species suggests that unique ecological, physiological, or genetic factors in these groups may have facilitated the repeated evolution of longevity, whereas shortened lifespan may arise more easily and in response to a variety of ecological pressures. These findings highlight the complex and dynamic evolutionary history of relative lifespans in mammals, demonstrating that both short-lived and long-lived lineages have independently and repeatedly arisen throughout the mammalian tree of life.

### Identification of candidate genes associated with relative lifespans in mammals

To identify candidate genes linked to mammalian relative lifespans, we obtained sequence alignments for one-to-one orthologous proteins across 122 mammalian species with available lifespan data (Additional file 1: Table S5) from the OrthoMaM database [25]. These species represent 18 orders and 57 families, covering 69.2% (18 out of 26) of the recognized mammalian orders [26] and exhibited LQ values ranging from 0.25 to 6.24, representing the wide diversity of mammalian relative lifespans (Additional file 2: Fig. S3). To ensure accurate estimates of evolutionary rates for proteins, we excluded artificial one-to-one orthologous sequences, poorly aligned fragments, and orthologous proteins represented by fewer than 50 species or shorter than 50 amino acids in alignment length. Finally, 15,231 high-quality one-to-one orthologous protein alignments were retained for further analysis. Based on the 122-species phylogeny derived from the OrthoMaM database [27], we performed Pearson correlation analyses between lifespan changes along phylogenetic branches and protein-specific relative evolutionary rates (RERs) using RERconverge [28]. We identified 396 genes with a significant negative correlation with lifespans (NCGs; Additional file 1: Table S6) and 232 genes with a significant positive correlation (PCGs; Additional file 1: Table S7).

**Fig. 3:**
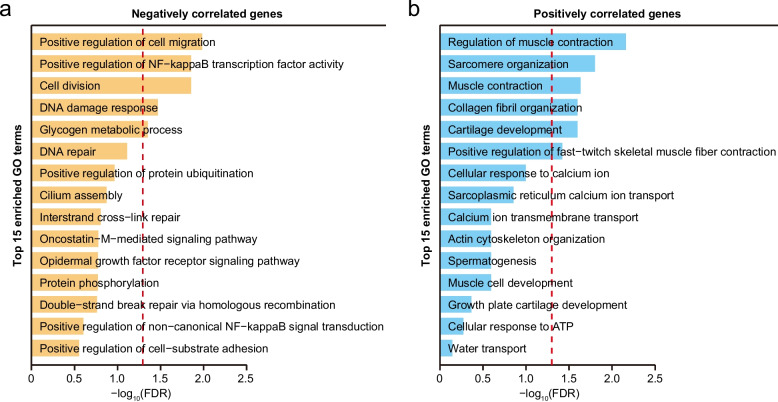
Functional enrichment analyses for genes correlated with mammalian relative lifespans. **a** The over-represented functional categories for genes that are negatively correlated with mammalian relative lifespans. **b** The over-represented functional categories for genes that are positively correlated with mammalian relative lifespans. The red dashed line indicates the threshold for statistical significance (FDR < 0.05)

To explore the biological functions of genes correlated with mammalian lifespan, we performed Gene Ontology (GO) enrichment analyses of NCGs (Additional file 1: Table S8). Of the top 15 enriched GO biological-process (BP) terms (Fig. 3a), there were 4 terms associated with DNA recombination and repair, such as DNA damage response (GO:0006974), DNA repair (GO:0006281), Interstrand cross-link repair (GO:0036297), and double-strand break repair (GO:0000724). There were two terms related to NF-κB pathways. These results were highly consistent with those observed in the previous study [1]. In addition to these consistent results, we found that the GO terms involving the genes related to cell migration and division were significantly enriched for the NCGs (FDR < 0.05; Fig. 3a). These results suggest that increased evolutionary constraint in the genes related to DNA repair, NF-κB pathways, and cell migration and division plays crucial roles for the evolution of mammalian lifespans. Similarly, we conducted GO enrichment analyses for the PCGs (Additional file 1: Table S9). Among the top 15 enriched GO BP terms, six were associated with muscle development and function, such as regulation of muscle contraction (GO:0006937; FDR = 0.0069) and sarcomere organization (GO:0045214; FDR = 0.016), and three were associated with calcium ion transport (Fig. 3b).

A decrease in relative evolutionary rates indicates stronger evolutionary constraints, suggesting that the NCGs are crucial in the evolution of long-lived mammalian species. This highlights the essential role of biological functions related to DNA repair, NF-κB pathways, and cell cycle in promoting mammalian longevity. By contrast, positive correlations indicate that genes with faster evolutionary rates in long-lived species than in short-lived ones may experience a relaxation of constraints or positive selection.

### Different evolutionary pressures on NCGs and PCGs

**Fig. 4:**
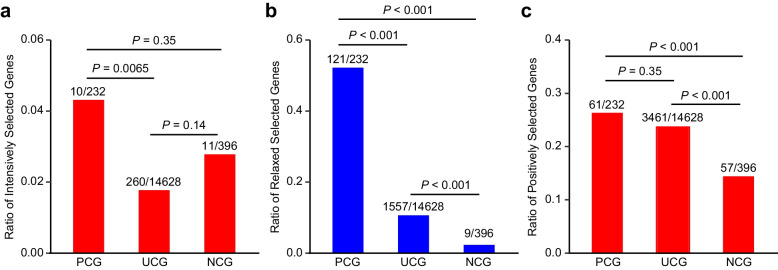
Examination of evolutionary pressures on NCGs and PCGs. **a** The proportion of intensively selected genes in relatively long-lived mammals is generally higher in NCGs compared to PCGs and UCGs. **b** The proportion of genes under relaxed selection in relatively long-lived mammals is significantly higher in PCGs than in NCGs and UCGs. **c** The proportion of positively selected genes in long-lived mammals is similar among PCGs, NCGs, and UCGs. The *P* values are from two-tailed *χ*^2^ tests

To further explore the evolutionary pressures on NCGs and PCGs, we applied the codon-based phylogenetic framework RELAX to determine whether selective strength was relaxed or intensified [29], which used 13 relatively long-lived species (LQ > 2.4) as foreground branches, while the remaining species were used as background branches in the 122 mammalian species. Among the 396 NCGs, 232 PCGs, and 14,628 uncorrelated genes (UCGs), 11, 10, and 260 genes, respectively, were found to be under intensified selection in relatively long-lived mammalian species (Additional file 1: Table S10). The proportion of PCGs (10/320 = 3.2%) under intensified selection was significantly higher than that of UCGs (260/14628 = 1.7%) (*P* = 0.0065, two-tailed *χ*^2^ test; Fig. 4a), and slightly higher than that of NCGs (11/396 = 2.7%). Conversely, in the relatively long-lived mammalian species, we identified 9 NCGs, 121 PCGs, and 1,557 UCGs under relaxed selection (Additional file 1: Table S11). The proportion of PCGs under relaxed selection (121/232 = 52.2%) was significantly larger than that observed for both NCGs (9/396 = 2.3%) and UCGs (1557/14628 = 10.6%) (*P* < 0.0001, *P* < 0.0001, two-tailed *χ*^2^ tests; Fig. 4b). Together, these results suggest that NCGs are more likely to experience intensified selection, while PCGs are more often subject to relaxed selection, highlighting the importance of both forms of selective pressure in shaping the evolutionary trajectories of mammalian lifespans.

To avoid arbitrary assignment of long-lived mammalian lineages, we used site models in PAML [30] to detect positive selection on NCGs and PCGs by comparing M8a (beta and ω_s_ = 1) with M8 (beta and ω). In this comparison, the null hypothesis M8a fixes ω_s_ at 1 for the site category ω_s_, whereas the alternative hypothesis M8 allows ω_s_ > 1. After this analysis, we found 57 NCGs, 61 PCGs, and 3461 UCGs under positive selection (Tables S12). The proportion of positively selected genes (PSGs) was significantly lower in NCGs (57/396 = 14.4%) compared to PCGs (61/232 = 26.3%) and UCGs (3461/14,628 = 23.7%) (*P* < 0.05, two-tailed *χ*^2^ tests; Fig. 4c). These results suggest that NCGs have experienced weaker positive selective pressure than PCGs and UCGs.

### In vivo* experiments to test the effects of PSGs associated with lifespan*

**Fig. 5:**
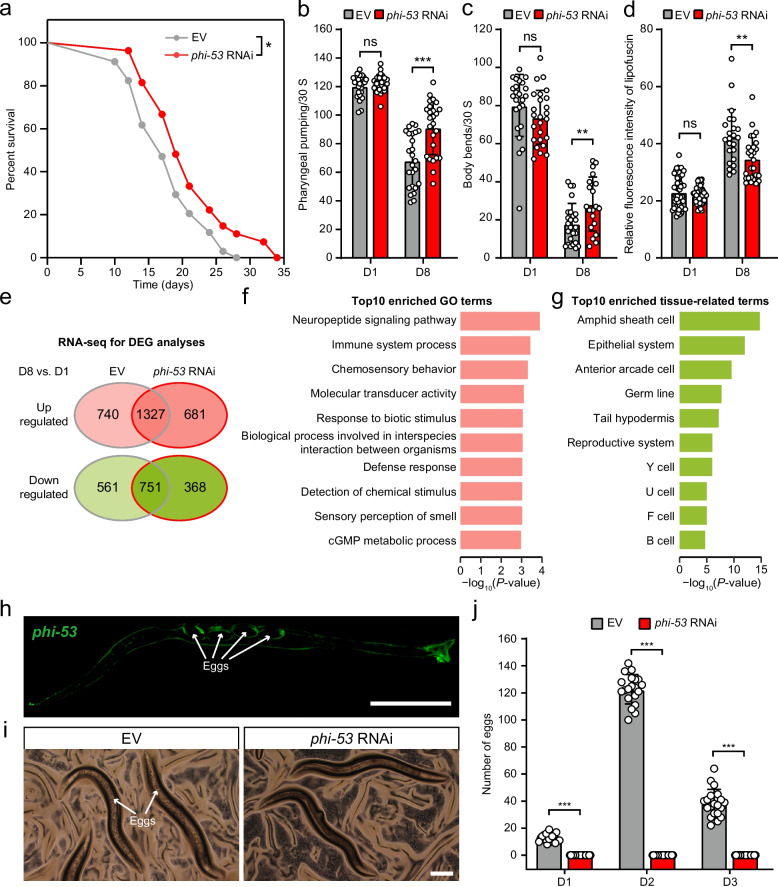
Knockdown of phi-53 extends lifespan in *C. elegans*. **a** Survival curves comparing worms treated with empty vectors (EV) to those treated with phi-53 RNAi. The *P* value is from the Mantel–Haenszel test. Knockdown of phi-53 delays the appearance of aging markers, including pharyngeal pumping (**b**), body bending (**c**), and lipofuscin (**d**). The *P* values are from the two-tailed Student’s *t*-tests. **e** Venn diagram showing significantly up-regulated and down-regulated genes between day 1 and day 8 in adult worms treated with either EV or phi-53 RNAi. **f** Top 10 enriched functional categories for genes specifically upregulated in 8-day-old worms subjected to phi-53 RNAi versus empty vector. **g** Top 10 enriched tissue-related terms for genes specifically downregulated in 8-day-old worms subjected to phi-53 RNAi versus empty vector. **h** Representative image of transgenic worms expressing phi-53p::phi-53::GFP. **i**–**j** Following phi-53 knockdown, *C. elegans* fails to produce eggs. Scale bars: 500 μm. The *P* values are from the two-tailed Student’s *t*-tests. The first day of adulthood for the worms is recorded as day 1 (D1)

The nematode *Caenorhabditis elegans* (*C. elegans*) serves as a well-established model for studying the genetic regulation of aging due to its short lifespan. To functionally validate our evolutionary analysis, we conducted lifespan assays in *C. elegans* to assess the effects of lifespan-associated PSGs on lifespan regulation. Among the PSGs in NCGs and PCGs, we found 16 high-confidence orthologs in *C. elegans* (Additional file 1: Table S13) based on gene annotations from the Ensembl database [31]. We focused our experiments on 11 genes that were present in the Ahringer RNAi library [32], which is available in our laboratory and had not been previously tested. Functional examinations revealed varying effects of these genes on lifespan regulation in *C. elegans*. After individual RNAi knockdown, *pri-1* (mammalian ortholog *PRIM1*), *rrp-8* (*RRP8*), *K07H8.2* (*SLC41A3*), *rfc-4* (*RFC4*), *C16C10.8* (*LYAR*), *C27A12.9* (*PIGO*), *K09E4.4* (*NAGLU*), *ZK185.2* (*SLC41A3*), and *ZK1053.6* (*SLC41A3*) showed no significant effect on the lifespan of *C. elegans* (Additional file 2: Fig. S4). Notably, RNAi knockdown of *Y45G12B.3* (*L2HGDH*) significantly reduced the lifespan of *C. elegans* (*P* = 0.026; Mantel–Haenszel test; Additional file 2: Fig. S4). In contrast, knockdown of *T24C4.5* (*PRIM1*), also known as *phi-53*, resulted in a significant increase in worm lifespan (*P* = 0.037; Mantel–Haenszel test; Fig. 5a). Furthermore, we assessed the impact of *phi-53* on various phenotypic traits associated with lifespan in worms [33], such as the pharyngeal-pumping rate, body bending, and the accumulation of lipofuscin autofluorescence. In 8-day-old worms, *phi-53* knockdown resulted in a significant increase in both the pharyngeal pumping rate and body bending (*P* < 0.01; two-tailed Student’s *t*-tests; Fig. 5b and c), along with a significant decrease in lipofuscin autofluorescence (*P* < 0.01; two-tailed Student’s *t*-tests; Fig. 5d) compared to controls. These findings strongly suggest that *phi-53* plays a crucial role in regulating *C. elegans* lifespan.

To investigate the mechanisms underlying *phi-53*-mediated longevity, we performed RNA-seq analysis to examine the transcriptional profiles of worms before and after *phi-53* knockdown. Principal component analysis showed that the gene expression profiles of worms treated with *phi-53* RNAi at days 1 and 8 of adulthood were similar to those treated with empty vectors (Additional file 2: Fig. S5), suggesting that *phi-53* knockdown did not substantially alter overall gene expression. However, we identified 2008 genes that were significantly up-regulated in 8-day-old *phi-53* RNAi worms compared to their 1-day-old counterparts (fold change > 5; FDR < 0.05). Similarly, 2067 genes were up-regulated in 8-day-old worms treated with the empty vector compared to 1-day-old worms. Of these, 1327 genes were common to both sets, suggesting a shared molecular response to aging. We then focused on the 681 genes that were specifically up-regulated following *phi-53* knockdown (Fig. 5e; Additional file 1: Table S14) and performed functional enrichment analyses using the WormBase Enrichment Suite [34] to gain insights into their biological implications. Among the significantly enriched functional categories (Additional file 1: Table S15), several Gene Ontology (GO) terms were linked to immune system process and responses to biotic stimulus (Fig. 5f). In addition to up-regulated genes, we found that 1119 genes were significantly down-regulated in 8-day-old worms compared to 1-day-old worms subjected to *phi-53* RNAi (Fold Change > 5; FDR < 0.05; Additional file 1: Table S15), while 2067 genes were up-regulated in 8-day-old worms compared to 1-day-old worms subjected to the empty vector. We further analyzed 368 genes that were specifically down-regulated following *phi-53* knockdown and conducted functional enrichment analyses (Additional file 1: Table S16). These down-regulated genes were notably enriched in categories related to the reproductive system and germ line (Fig. 5g). To validate this finding, we generated a transgenic *C. elegans* line expressing *phi-53p::phi-53::GFP*, which showed *phi-53* expression in the eggs and cuticle of the worms (Fig. 5h). Furthermore, knockdown of *phi-53* severely impaired the worm’s egg-laying ability (*P* < 0.001; two-tailed Student’s *t*-tests; Fig. 5i and j). Together, based on the survival curve, worms with *phi-53* knockdown had a lifespan of approximately 35 days, which is significantly longer than the average worm lifespan (Fig. 5a). This suggests that *phi-53* plays an important role in regulating lifespan. Consistent with this, the observed downregulation of reproductive system genes suggests that *phi-53* may influence lifespan through the classical reproduction-soma trade-off mechanism [35]. Additionally, the upregulation of immune-related genes following *phi-53* knockdown suggests that it could also regulate healthspan by enhancing immune and stress response pathways, thereby improving resistance to biotic stressors and supporting the maintenance of physiological function. These findings largely support the hypothesis that suppressing genes involved in the reproductive system can extend the lifespan of *C. elegans* by reallocating resources from reproduction to maintenance and repair [36].

## Discussion

In this study, we revealed that phylogenetic relationships across mammalian species significantly influence the evolutionary patterns of lifespans at broad phylogenetic scales. The examination of evolutionary models suggested that the evolution of mammalian lifespans relatively stabilized along the ancestral branches but fluctuated rapidly at the terminal branches across the phylogeny of mammals. Previous studies mapping maximum lifespans onto mammalian phylogenies showed that closely related species tend to have similar lifespans [9]. This signal is most pronounced along deep, ancestral branches, and certain mammalian clades, such as primates and bats, often exhibit clade-wide constraints on longevity that have persisted since the origin of the clades [5, 13]. Deep branches are shaped by long-term ecological and physiological constraints, which change slowly over evolutionary time due to stabilizing selection and functional trade-offs [4]. In contrast, more rapid and larger fluctuations in maximum lifespan occurring in terminal branches or in the recent history of individual lineages may be responsible for ecological or behavioral innovations (e.g., lower predation, new metabolic/physiological adaptations), which relax previous constraints and allow rapid (sometimes convergent) evolution of lifespan traits [37, 38].

The strong phylogenetic signal in the evolution of mammalian lifespans indicates that analyses of this trait cannot assume species are phylogenetically independent. To avoid simplified estimations and biased associations between genetic changes and mammalian lifespans, it is crucial to assess and incorporate the most suitable evolutionary model. Compared to the previous study that identified lifespan-associated genes by correlating relative evolutionary rates with lifespans across 61 mammals [1], our analysis incorporated 81 additional unique species, spanning 61 genera, 22 families, and 3 orders. This expansion significantly increases taxonomic coverage and enables a more robust correlation analysis across the mammalian phylogeny. To enhance the precision of our evolutionary rate estimates, we took special care in accurately identifying one-to-one orthologous genes. Importantly, our analyses encompass genes both negatively and positively correlated with lifespan, indicating that genes undergoing accelerated evolution may also play important roles in mammalian lifespan evolution. Among the candidate genes identified, those subject to strong evolutionary constraints in long-lived mammalian species were significantly enriched in functional categories related to DNA repair. This finding aligns with previous studies [1, 2, 14, 15, 39], supporting the reliability of our analyses and highlighting the crucial roles of DNA repair in the evolution of mammalian lifespans. In addition to confirming previously identified functional categories, our gene enrichment analyses also revealed new GO terms, including those related to cell migration and cell division, that are significantly associated with lifespan-related genes.

Notably, our analyses focused not only on genes under purifying selection in long-lived mammalian species but also on genes evolving more rapidly and under relaxed selection in these species. These relaxed genes were enriched in functional categories related to calcium ion transport, which is closely linked to neuronal calcium ion homeostasis and excitability [40]. Notably, genes associated with neurotransmission are significantly downregulated in exceptionally long-lived humans, and reducing excitability with a Ca^2+^ channel blocker has been shown to extend the lifespan of *C. elegans* [41]. On the other hand, we verified the impact of PSGs in long-lived mammals on lifespans using in vivo experiments. The results provide insights into how genetic inactivation of *phi-53* affects the balanced modulation of immune and reproductive systems, which underlie lifespan extension in *C. elegans*. Because the specific biological functions of this gene remain largely uncharacterized, future studies are warranted to investigate its potential roles, particularly in cell division and DNA repair, to further elucidate its contribution to lifespan variation. In addition to the 11 genes examined, five PSGs in our dataset—*Y119D3B.14*, *ddo-1*, *ddo-2*, *ddo-3*, and *nol-2*—have attracted attention due to their diverse biological functions. Y119D3B.14 is predicted to possess GTPase activity and to participate in mitochondrial translation and ribosome disassembly, though its precise physiological role remains unclear [42]. The genes *ddo-1*, *ddo-2*, and *ddo-3* encode distinct isoforms of D-aspartate oxidase, which are involved in the catabolism of D-amino acids [43]. In contrast, *nol-2* encodes a methyltransferase that negatively regulates locomotion and positively regulates reproductive processes [44]. These studies highlight the functional diversity of PSGs within our dataset and suggest broad biological implications for these genes. Upregulating genes involved in immune responses can enhance an organism’s ability to defend against pathogens and environmental stressors, potentially increasing survival and longevity [45]. However, allocating resources to heightened immune function can come at the expense of reproductive capacity. Our findings support the principle of life-history trade-offs between immune function and other energetically expensive processes [46], although future studies are needed to explore the underlying regulatory mechanisms in light of these results.

Our study used two comprehensive datasets for large-scale comparative analyses of mammalian lifespans and the genes associated with them. The broad taxonomic coverage of these datasets enhances the generalizability of our findings and reduces taxon sampling bias, thereby increasing the robustness of comparative studies across the mammalian phylogeny [47]. Nevertheless, the completeness and quality of species representation remain vital considerations. Although our datasets encompass the majority of extant mammalian orders and families, certain lineages are underrepresented or missing. This uneven sampling can introduce phylogenetic gaps, potentially overlooking specific evolutionary events or adaptations, and may lead to biased estimates of phylogenetic signal or evolutionary rates [48]. Further, our analyses depend on phylogenetic trees derived from widely used public resources such as the TimeTree [19] and OrthoMam [27] databases. While these resources offer extensive coverage, they also have limitations. Many nodes—especially those corresponding to deep divergences or rapid radiations—are poorly resolved due to incomplete or ambiguous molecular data. Such topological uncertainties can affect downstream analyses that require precise tree structure, including the estimation of phylogenetic signal, the inference of trait evolution, and the detection of lineage-specific rate shifts [49]. Together, although the unprecedented breadth of our datasets maximizes the scope and robustness of our evolutionary analyses, limitations in taxon representation and phylogenetic tree certainty could still affect the reliability of specific results. Recognizing these sources of uncertainty is crucial, and ongoing improvements in both taxon sampling and phylogenetic inference will continue to enhance the rigor and reliability of investigations into the evolutionary mechanisms of mammalian lifespans.

## Conclusions

Mammalian lifespan shows remarkable diversity, but its evolutionary history and molecular basis remain poorly understood. By integrating large-scale phylogenetic reconstruction with comparative genomics, our study identifies key genetic and evolutionary factors underlying lifespan variation across mammals. Our findings reveal how shared evolutionary history shapes lifespan, pinpoint genes and pathways involved in longevity and aging, and validate their functional roles in vivo. These results provide valuable molecular insights and establish a comprehensive framework for understanding the evolution and regulation of lifespan in mammals.

## Methods

### Lifespan and body mass data in mammals

Data for the maximum lifespan and the averaged adult body mass of mammalian species were sourced from the AnAge database (version: Build 15) [17]. To ensure the reliability of the data, we excluded species whose lifespan estimates were based on a very small sample size (< 10 specimens). We also excluded species not present in the TimeTree database [19] as their phylogenetic relationships are necessary for subsequent evolutionary analyses. After filtering, a total of 968 mammalian species were obtained, which spanned 24 orders and 122 families of mammals and accounted for 85.7% (24/28) of extant mammalian orders and 78.2% (122/156) extant mammalian families (Additional file 1: Table S1). Because lifespan is closely correlated with body mass, the longevity quotient (LQ) is commonly used in comparative biology to assess lifespan differences across species [9]. LQ is calculated as the ratio of a species’ observed maximum lifespan to its expected maximum lifespan [50]. According to the AnAge database documentation (http://genomics.senescence.info/help.html#anage), the expected maximum lifespan for each mammalian species is estimated using a least-squares regression formula: *t*_max_ = 4.88 × *M*^0.153^, where *t*_max_ is the expected maximum lifespan (in years) and *M* is the adult body mass (in grams). Species with LQ < 1 means that the species’ observed maximum lifespan is less than the expected maximum lifespan, indicating it is relatively short-lived, while those with LQ > 1 are deemed relatively long-lived.

### Evolutionary models for testing macroevolution of mammalian lifespans

To investigate the evolution patterns underlying mammalian lifespans, we tested several evolutionary models using LQ values from 968 mammalian species, employing the *R* package geiger (v2.0.11) [18]. The models assessed included white-noise, Brownian motion, Pagel’s lambda (λ), kappa (κ), delta (δ), Ornstein–Uhlenbeck, early-burst, trend diffusion, and mean trend. These models are commonly used in studies of macroevolutionary patterns in phylogenetic traits and together encompass a wide range of possible evolutionary scenarios [18, 51]. The theoretical basis and assumptions of each evolutionary model, along with the corresponding references, were provided in a supplementary table (Additional file 1: Table S17). This comprehensive modeling approach increases our ability to identify the best-fitting evolutionary mode for mammalian lifespans. The best-fitting model was determined by the highest log-likelihood and the lowest AIC, following standard model selection procedures [51].

### Identification of genes correlated with mammalian lifespans

Sequence alignments of one-to-one orthologous genes from 190 mammalian species were retrieved from the OrthoMaM database (version 12) [27]. To ensure the accuracy of the orthologous sequences, we used human and mouse protein sequences from the UCSC database [52] as references. Each sequence underwent a BLAST search against the one-to-one orthologous genes, and any gene whose best hit did not align with the human or mouse reference sequences was excluded from the dataset. We also refined the dataset by eliminating poorly aligned regions using trimAL [53] with the parameters “-resoverlap 0.70 -seqoverlap 50-automated1 -colnumbering”. We retained only orthologous alignments that contained sequences from at least 50 species and were at least 50 amino acids in length. To ensure broad taxonomic representation, we restricted our analysis to orthologous alignments containing sequences from at least 50 species. Lowering this threshold resulted in the loss of key representative taxa, potentially biasing the comparative genomic analysis. Small proteins are typically defined as those consisting of fewer than 50 amino acids [54, 55]; thus, by applying this length cutoff, we aimed to minimize errors in orthologous alignments associated with shorter sequences. Of the 190 mammals with one-to-one orthologous genes available in the OrthoMaM database, 122 species had available lifespan and body mass data in the AnAge database. The median, mean, and variance for both lifespan and body mass across all orders represented in the 122 species were provided (Additional file 1: Table S18). These metrics were closely aligned with the corresponding values from the > 900 mammalian species initially analyzed (Additional file 1: Table S2), ensuring no significant sampling bias. Consequently, these 122 species were used to estimate correlations between lifespan and the relative evolutionary rates of proteins.

We first obtained the phylogenetic relationships among the 122 mammalian species from the OrthoMaM database. For each of the 15,231 one-to-one orthologous genes, we extracted the corresponding subset of the species tree based on the species present for that gene using the newick-utils package (v1.6) [56]. We then calculated the correlation between the evolutionary changes in the LQ value and the relative evolutionary rates of each one-to-one orthologous gene across the 122 species using the *R* package RERconverge (v0.1.0) [28]. Briefly, this method quantifies RERs of each protein by measuring its rate deviation along specific phylogenetic branches compared to proteome-wide expectations. To estimate the correlation between RERs and the evolution of mammalian lifespans, we calculated lifespan changes along phylogenetic branches using maximum-likelihood ancestral-state reconstruction [22]. We used the char2Paths() function based on the species tree to generate a lifespan vector, representing the predicted difference in lifespans between each species and its ancestor. We then applied the getAllCor() function to perform Pearson correlation analyses between these transformed lifespan values and the RER matrix. To control for false discovery rates, RERconverge developed the phylogenetically restricted strategy [28]. Specifically, RERconverge repeatedly simulates lifespan values for each species along the phylogeny under a Brownian motion model, then reassigns the original lifespan values to species based on the simulated rank order. After each simulation, it recomputes the correlation between gene evolutionary rates and lifespan. A total of 1000 such iterations were performed to obtain empirical *p*-values. This approach by RERconverge is equivalent to phylogenetically independent contrasts, effectively removing phylogenetic dependence between gene evolutionary rates and lifespans across the mammalian phylogeny [28].

### Examining evolutionary pressures on genes correlated with mammalian lifespans

Since over 400 species in our dataset have LQ > 1, we set a more stringent threshold (LQ > 2.4) to identify exceptionally long-lived species and minimize false positives when detecting selection pressure on genes acting on relatively long-lived mammals. We set the LQ cutoff of 2.4 for relatively long-lived lineages, as only about 5% of mammalian species in our dataset had LQ values above this threshold. To examine the evolutionary pressures on long-lived mammalian species, we used the RELAX tool [29] to estimate the distribution of the nonsynonymous to synonymous substitution ratio (ω) for each one-to-one orthologous gene in 16 long-lived mammals (LQ > 2.4) among 122 mammalian species. RELAX compares two subsets of branches in a phylogeny to determine whether selective strength is relaxed or intensified in one subset relative to the other. If the parameter *k* was significantly larger than 1, it indicated intensified selection on the gene; if *k* was significantly less than 1, it suggested relaxed selection. Additionally, to avoid arbitrarily assigning long-lived mammalian lineages, we used site models in PAML [57] to detect positive selection on genes correlated with mammalian lifespans. Specifically, we compared model M8a (beta and ω_s_ = 1) with M8 (beta and ω), where the null hypothesis M8a fixes ω_s_ at 1 for the site category ω_s_, while the alternative hypothesis M8 allows ω_s_ > 1. This comparison has been demonstrated to be more robust than other site model tests (e.g., M1a vs. M2a; M7 vs. M8) [58, 59]. Both methods utilized maximum likelihood estimation to determine whether the alternative hypothesis was supported over the null hypothesis of no significant difference. We assessed statistical significance using a two-tailed *χ*^2^ test. The intensified selection estimated using RELAX indicates a general strengthening of selection, which may reflect either stronger purifying selection, stronger positive selection, or both. In contrast, PAML specifically detects signals of positive selection at particular sites or branches.

### Functional enrichment analyses

We used the clusterProfiler [60] to identify overrepresented Gene Ontology Biological Process (GO BP) terms among genes significantly correlated with mammalian lifespan. The positively correlated genes (PCGs) and negatively correlated genes (NCGs) were used as the foreground gene sets, respectively, while all one-to-one orthologous genes served as the background gene sets. We excluded overrepresented GO BP terms associated with more than 300 background genes, as these terms usually represent broader GO BP levels and provide limited specific insights. In addition, we employed REVIGO [61] to filter out redundant significant GO BP terms, using a medium cutoff of 0.7 for allowed similarity. This approach helped refine the analysis by focusing on more specific and informative biological processes.

### RNA interference for worms

WT worms were the *C. elegans* Bristol N2 strain and were grown on nematode growth media (NGM) agar plates seeded with *E. coli* strain OP50 at 20 °C. RNA interference (RNAi) bacterial strains targeting specific genes were obtained from the Ahringer RNAi library [32], and all clones used in this study were verified through sequencing. To prepare these strains, *E. coli* HT115 expressing dsRNA was cultured overnight at 37 °C in LB (Luria–Bertani) medium supplemented with 100 μg/ml ampicillin. These cultures were then spread onto NGM plates containing 100 μg/ml ampicillin and 5 mM isopropyl 1-thio-β-D-galactopyranoside (IPTG). The RNAi-expressing bacteria were incubated at 25 °C overnight. Synchronized L1 larvae were placed on these plates and maintained at 20 °C until they matured into young adult worms. To validate RNAi efficiency, we performed RT-qPCR for 11 candidate genes and confirmed that *E. coli* strains from the Ahringer RNAi library successfully knocked down their expression (Additional file 2: Fig. S6).

### Transcriptome sequencing and analysis for *C. elegans*

One-day-old (D1) or 8-day-old (D8) adults subjected to either *phi-53* RNAi or exposed to the control L4440 RNAi (empty vector) were collected. These worms were washed off the plates using M9 solution, followed by two additional washes using M9 buffer. Three biological-replicate samples were collected on different days. Total RNA was extracted using the Qiagen RNeasy Mini Kit, and the RNA was eluted in RNase-free water. The quality and integrity of RNA samples were assessed using an Agilent 2100 Bioanalyzer. Approximately 5 μg of total RNA from each replicate was used to construct cDNA libraries. Sequencing was performed on the Illumina HiSeq 2000 platform, generating 100-nucleotide paired-end reads according to the manufacturer’s guidelines, with each library achieving a sequencing depth of 5–10 Gb. The raw RNA-seq reads were first cleaned with fastp [62], then trimmed with Trimmomatic [63] using default parameters, and aligned to the *C. elegans* WBcel235 reference genome (GCF_000002985.6) with HISAT2 [64]. The transcriptome was assembled with StringTie [65], and feature counts were generated with featureCounts [66]. Differentially expressed genes were identified using a two-tailed Student’s *t*-test. Genes with significantly differential expression were classified into functional categories using the WormBase Enrichment Suite [34].

### Lifespan assays

To test the roles of 11 candidate genes in *C. elegans* lifespan, we conducted experiments using synchronized young adult *C. elegans* populations. These worms were cultured on NGM plates inoculated with *E. coli* OP50 at 20 °C [33]. The first day of adult life was designated as day 1. During the reproductive phase, worms were transferred daily to fresh plates. After reproduction ended, they were transferred every three days. We recorded the number of living worms daily, considering worms dead if they failed to move upon gentle prodding and showed no pharyngeal pumping. Each lifespan assay included three independent experiments, each with more than 50 worms.

### Age-related phenotypic marker assays

To assess age-related phenotypes in *C. elegans*, we examined three specific traits in both 1-day-old and 8-day-old worms, as in our previous study [67]. Briefly, pharyngeal pumping was measured by counting the number of contractions in the terminal bulb of the pharynx over 30-s intervals. Body bending activity was assessed by counting the number of body bends within 30 s. Each assay involved 30–50 worms and was repeated in three independent experiments. To measure lipofuscin, worms were mounted onto microscope slides, and images were captured using bright-field and 4′,6-diamidino-2-phenylindole (DAPI) channels. The blue autofluorescence intensity in the DAPI images was analyzed using ImageJ [68]. At least 50 worms were examined per assay, also in three independent experiments.

### Genetic manipulation of *C. elegans*

For gene overexpression, the *phi-53* gene, including its native promoter, was cloned into the vector pPD95.75 in frame with GFP using the infusion cloning method. Germline transformation was performed by microinjecting the prepared DNA constructs at 40 ng/μL into the N2 strain of *C. elegans* by standard techniques. The transgenic worms were confirmed before the assays.

## Supplementary Information

Additional File 1: Tables S1-S18. Table S1. Lifespan and body mass values of 968 mammalian species from the AnAge database. Table S2. Lifespans of different mammalian orders. Table S3. Coefficient of variability of LQ values at the mammalian family level. Table S4. Summary of model fitting for absolute mammalian lifespans. Table S5. 122 mammalian species used for detecting lifespan-associated genes. Table S6. Negatively correlated genes between relative evolutionary rate and lifespan. Table S7. Positively correlated genes between relative evolutionary rate and lifespan. Table S8. The over-represented GO Biological Process terms of negatively correlated genes. Table S9. The over-represented GO Biological Process terms of positively correlated genes. Table S10. Correlated genes under intensified selection in long-lived mammals. Table S11. Correlated genes under relaxed selection in long-lived mammals. Table S12. List of positively selected genes. Table S13. High-confidence orthologous PSGs between C. elegans and mammals. Table S14. Up and down-regulated genes before and after inhibiting phi-53 in *C. elegans*. Table S15. Enriched functional terms for the up-regulated genes after inhibiting phi-53 in *C. elegans*. Table S16. Enriched functional terms for the down-regulated genes after inhibiting phi-53 in *C. elegans*. Table S18. Summary of lifespan and body mass values of 122 mammalian species.

Additional File 2: Figures S1-S6. Fig. S1. The distribution of LQ valuesand log-transformed LQ valuesacross 968 mammalian species. Fig. S2. Ancestral state reconstruction of longevity quotientfor 968 mammalian species. Lineages with LQ values below 0.4 were classified as exceptionally short-lived, while those with LQ values above 2.4 were classified as exceptionally long-lived. Fig. S3. The phylogenetic tree of 122 mammalian species with their corresponding LQ values. Fig. S4. Survival curves comparing worms treated with empty vectorsto those treated with the target gene RNAi. The *P* values are from the Mantel–Haenszel tests. Fig. S5. PCA plot of transcriptomic data for the worms treated with EV and phi-53 RNAi at day 1 and day 8. Fig. S6. Validation of RNAi efficiency via RT-qPCR. RNAi was initiated at the L4 larval stage, and transcript levels were quantified on the third day of adulthood. Nematodes fed with an empty vectorserved as the control group. Relative expression levels were normalized to the reference genes act-1 and cdc-42 and are presented relative to the EV control. Data represent the mean of three to four independent biological replicates, with each sample measured in technical triplicate. Error bars indicate the standard error of the mean. Statistical significance was determined using a two-tailed Student’s *t*-test.

## Data Availability

The datasets supporting the conclusions of this article are available in the National Center for Biotechnology Information Sequence Read Archive database (BioProject: PRJNA1190120).

## Abbreviations

LQ: Longevity quotient
NGM: Nematode growth medium
DAPI: 4′,6-Diamidino-2-phenylindole
GFP: Green fluorescent protein
IPTG: Isopropyl 1-thio-β-D-galactopyranoside
PAML: Phylogenetic Analysis by Maximum Likelihood
