# Supplementary material for: Reconstructing mammalian lifespan evolution reveals strong phylogenetic effects and lifespan-associated genes
Source: BMC Biol. 2026 Apr 17;24:127. doi: 10.1186/s12915-026-02599-3 (PMC13217714; doi:10.1186/s12915-026-02599-3)
Supplement: Supplementary file 2 — Additional File 2: Figures S1-S6. Fig. S1. The distribution of LQ valuesand log-transformed LQ valuesacross 968 mammalian species. Fig. S2. Ancestral state reconstruction of longevity quotientfor 968 mammalian species. Lineages with LQ values below 0.4 were classified as exceptionally short-lived, while those with LQ values above 2.4 were classified as exceptionally long-lived. Fig. S3. The phylogenetic tree of 122 mammalian species with their corresponding LQ values. Fig. S4. Survival curves comparing worms treated with empty vectorsto those treated with the target gene RNAi. The P values are from the Mantel–Haenszel tests. Fig. S5. PCA plot of transcriptomic data for the worms treated with EV and phi-53 RNAi at day 1 and day 8. Fig. S6. Validation of RNAi efficiency via RT-qPCR. RNAi was initiated at the L4 larval stage, and transcript levels were quantified on the third day of adulthood. Nematodes fed with an empty vectorserved as the control group. Relative expression levels were normalized to the reference genes act-1 and cdc-42 and are presented relative to the EV control. Data represent the mean of three to four independent biological replicates, with each sample measured in technical triplicate. Error bars indicate the standard error of the mean. Statistical significance was determined using a two-tailed Student’s t-test. [file 12915_2026_2599_MOESM2_ESM.pdf]

Fig. S1. The distribution of LQ values (A) and log-transformed LQ values (B) across 968 mammalian species.

Fig. S2. Ancestral state reconstruction of longevity quotient (LQ) for 968 mammalian species. Lineages with LQ values below 0.4 were classified as exceptionally short-lived (shown in blue), while those with LQ values above 2.4 were classified as exceptionally long-lived (shown in red).

Fig. S3. The phylogenetic tree of 122 mammalian species with their corresponding LQ values.

Fig. S4. Survival curves comparing worms treated with empty vectors (EV) to those treated with the target gene RNAi. The *P* values are from the Mantel-Haenszel tests.

Fig. S5. PCA plot of transcriptomic data for the worms treated with EV and phi-53 RNAi at day 1 and day 8.

Fig. S6. Validation of RNAi efficiency via RT-qPCR. RNAi was initiated at the L4 larval stage, and transcript levels were quantified on the third day of adulthood. Nematodes fed with an empty vector (EV) served as the control group. Relative expression levels were normalized to the reference genes act-1 and cdc-42 and are presented relative to the EV control. Data represent the mean of three to four independent biological replicates (each containing four nematodes), with each sample measured in technical triplicate. Error bars indicate the standard error of the mean (SEM). Statistical significance was determined using a two-tailed Student's *t*-test (**P* < 0.05, ***P* < 0.01).

Fig. S1

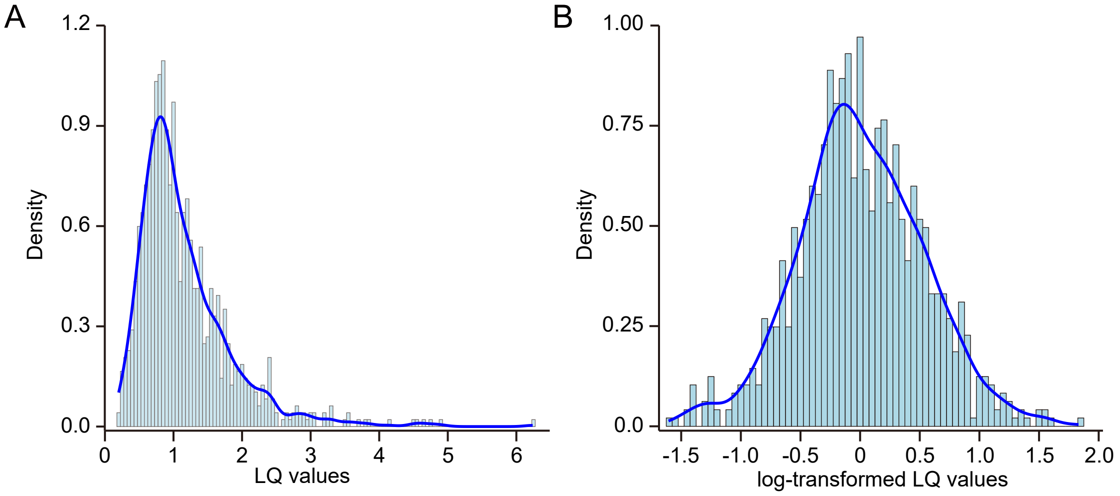

A
B
Density
LQ values
log-transformed LQ values
0.0
0.3
0.6
0.9
1.2
0
1
2
3
4
5
6
0.00
0.25
0.50
0.75
1.00
-1.5
-1.0
-0.5
0
0.5
1.0
1.5
2.0

Fig. S2

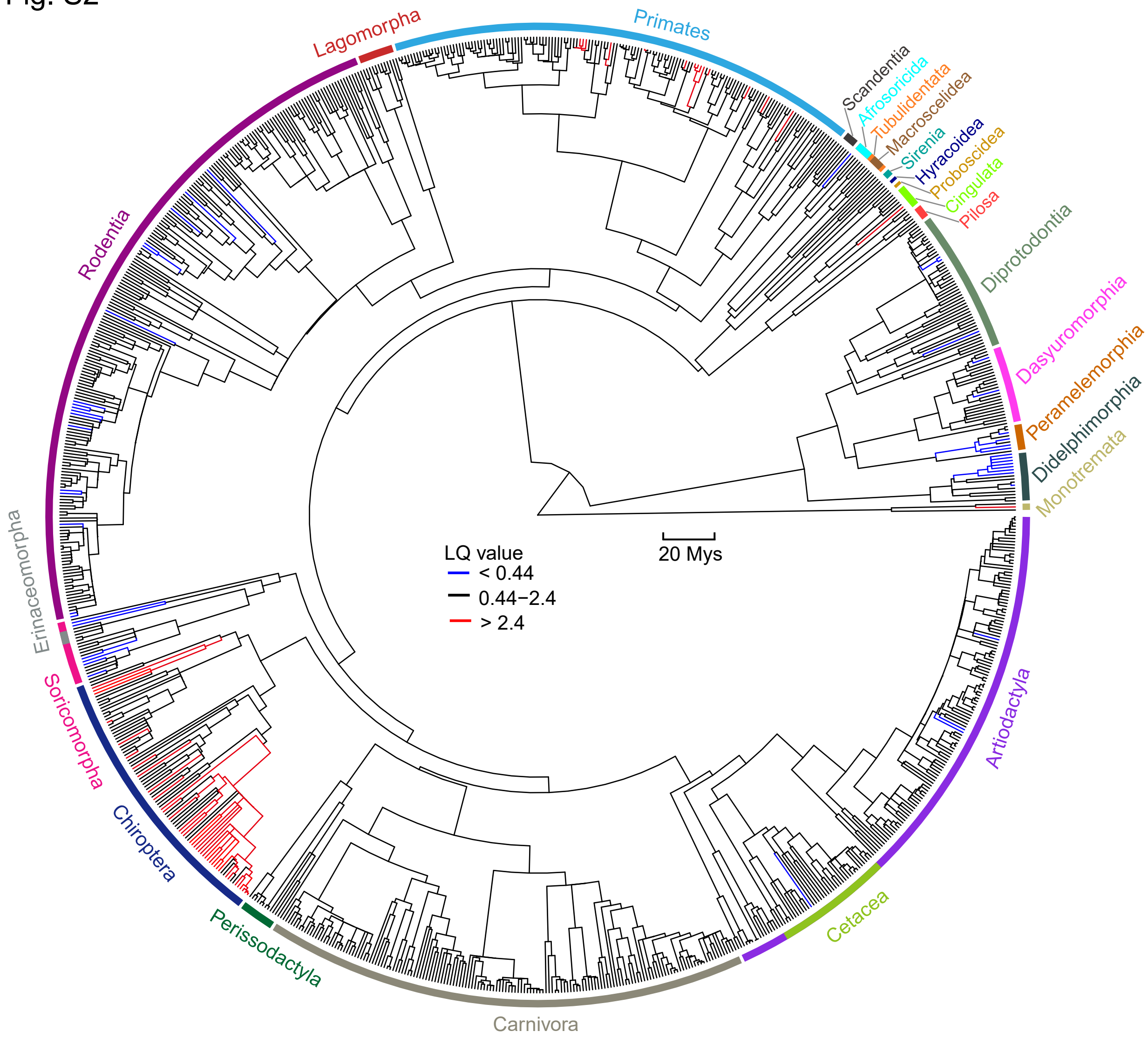

Primates
Lagomorpha
Rodentia
Scandentia
Afrosoricida
Tubulidentata
Macroscelidea
Sirenia
Hyracoidea
Proboscidea
Cingulata
Pilosa
Diprotodontia
Dasyuromorphia
Peramelemorphia
Didelphimorphia
Monotremata
Artiodactyla
Cetacea
Carnivora
Perissodactyla
Chiroptera
Soricomorpha
Erinaceomorpha
LQ value
< 0.44
0.44−2.4
> 2.4
20 Mys

Fig. S3

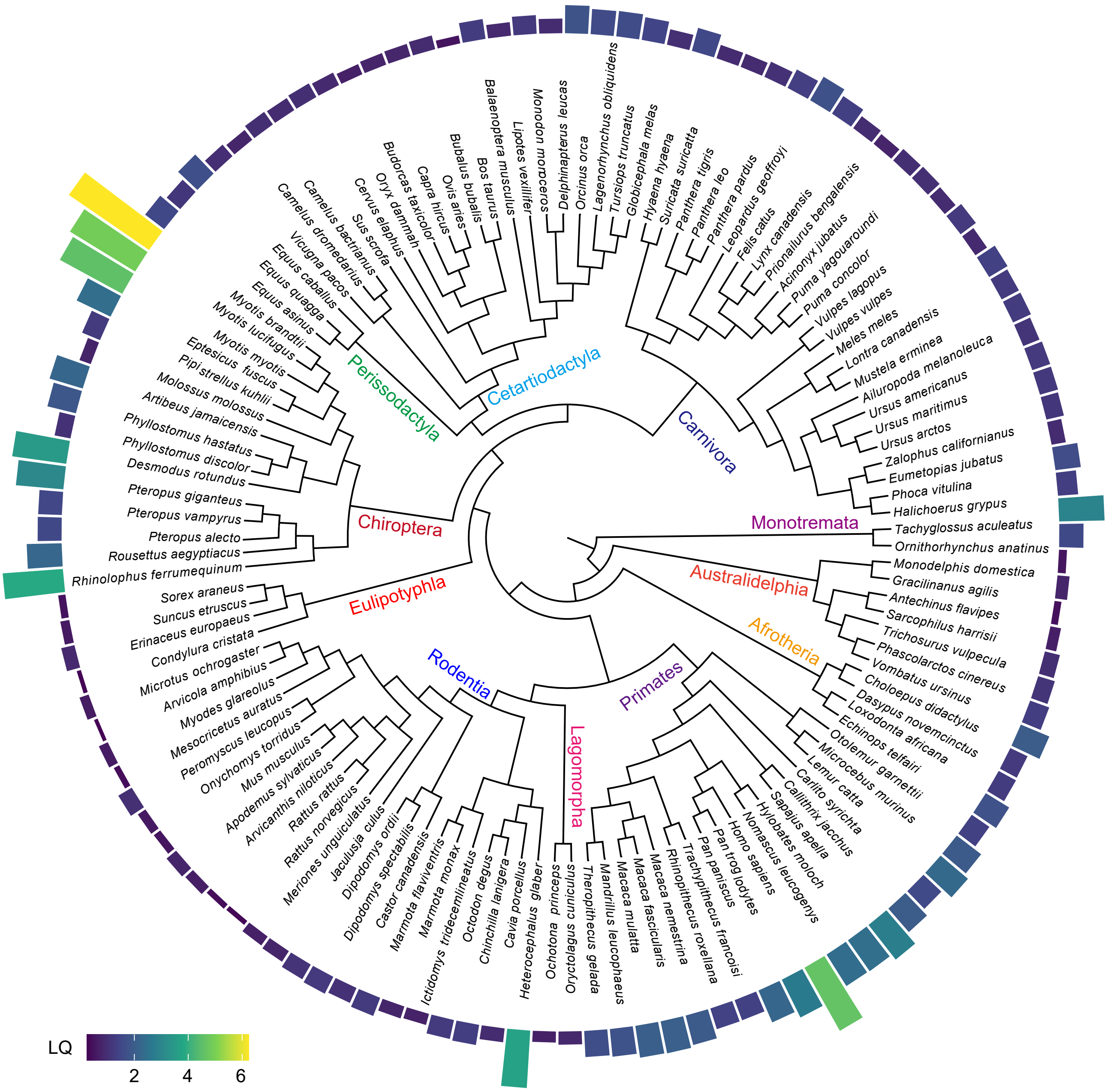

Cetartiodactyla
Perissodactyla
Carnivora
Chiroptera
Monotremata
Australidelphia
Afrotheria
Eulipotyphla
Rodentia
Lagomorpha
Primates
LQ
2
4
6

Fig. S4

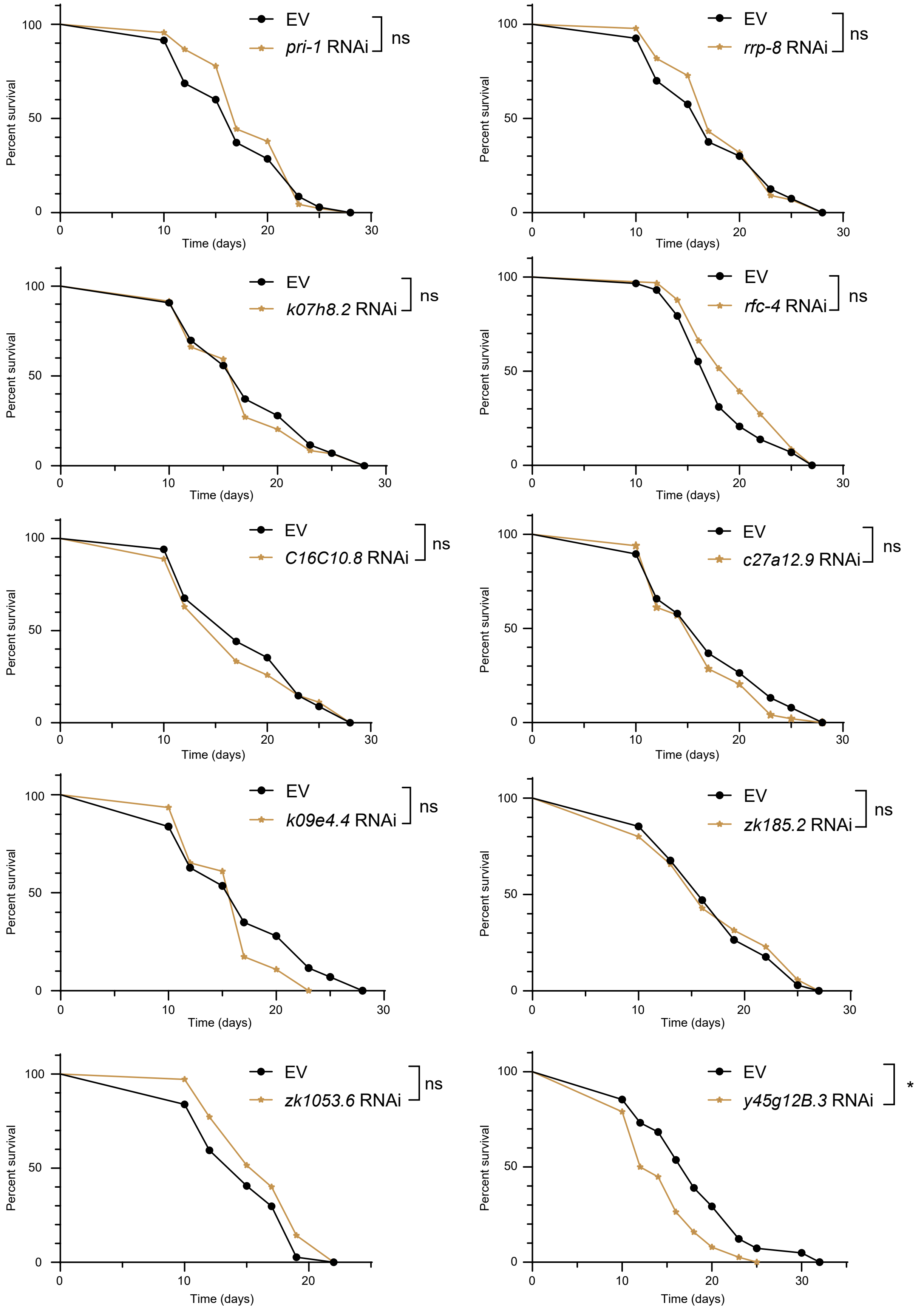
EV
pri-1 RNAi
ns
EV
rrp-8 RNAi
ns
EV
k07h8.2 RNAi
ns
EV
rfc-4 RNAi
ns
EV
C16C10.8 RNAi
ns
EV
c27a12.9 RNAi
ns
EV
k09e4.4 RNAi
ns
EV
zk185.2 RNAi
ns
EV
zk1053.6 RNAi
ns
EV
y45g12B.3 RNAi
*
Percent survival
Time (days)

Fig.S5

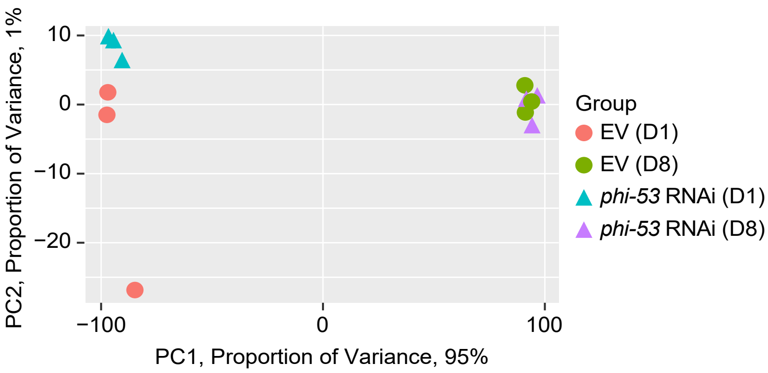

PC2, Proportion of Variance, 1%
10
0
−10
−20
−100
0
100
PC1, Proportion of Variance, 95%
Group
EV (D1)
EV (D8)
phi-53 RNAi (D1)
phi-53 RNAi (D8)

Fig. S6

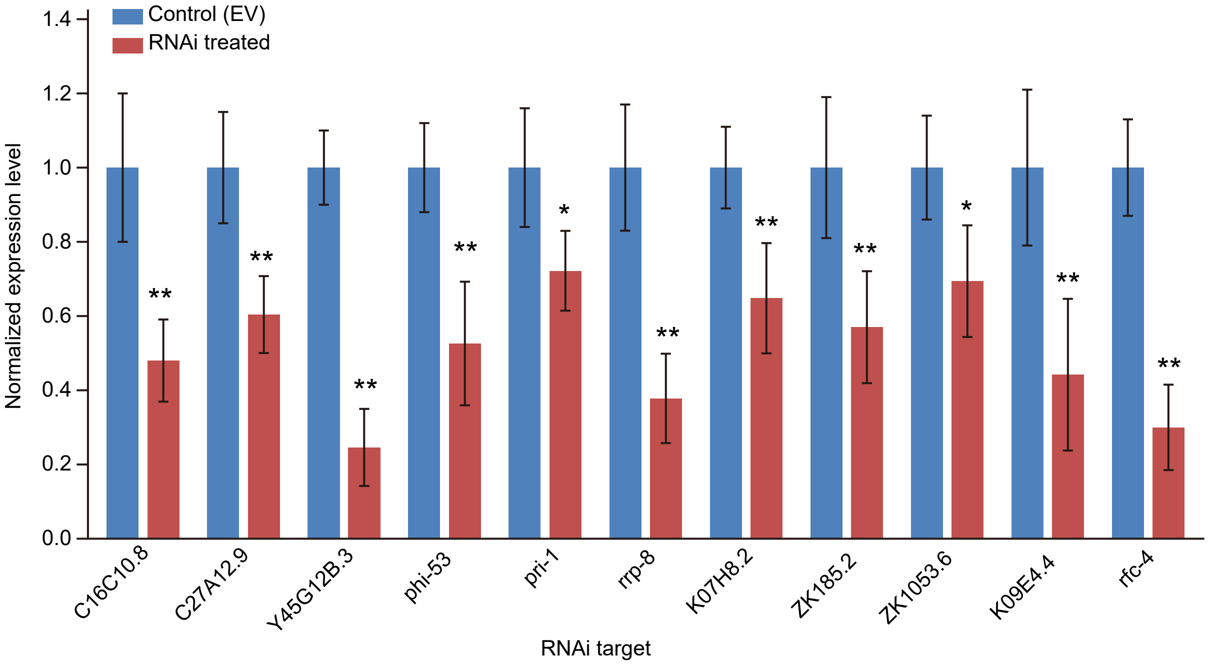

Control (EV)
RNAi treated
Normalized expression level
1.4
1.2
1.0
0.8
0.6
0.4
0.2
0.0
**
**
**
**
*
**
**
**
*
**
**
C16C10.8
C27A12.9
Y45G12B.3
phi-53
pri-1
rrp-8
K07H8.2
ZK185.2
ZK1053.6
K09E4.4
rfc-4
RNAi target
